# Olfactory abnormalities in patients with migraine: a narrative review of a symptom commonly overlooked by neurologists

**DOI:** 10.22514/jofph.2025.065

**Published:** 2025-12-12

**Authors:** Dong-xue Ren, Mao-mei Song, Ying-jie Gao, Chang-xin Li, Sui-yi Xu

**Affiliations:** ^1^Department of Neurology, Headache Center, The First Hospital of Shanxi Medical University, 030001 Taiyuan, Shanxi, China; ^2^Department of Neurology, Headache Center, Tianjin First Central Hospital, 300190 Tianjin, China

**Keywords:** Migraine, Osmophobia, Olfactory hallucinations, Hyperosmia, Hyposmia, Anosmia

## Abstract

Nausea, vomiting, photophobia, and phonophobia are common concomitant symptoms in patients with migraine and provide valuable information for headache specialists during consultations. Clinical observations have identified various olfactory abnormalities in patients with migraine, such as osmophobia, olfactory hallucinations, hyperosmia and hyposmia, which are often overlooked by neurologists. These olfactory abnormalities may interact with the trigeminal vascular system, parasympathetic nervous system, and cortical spreading depression. This review aimed to examine the mechanisms underlying olfactory abnormalities in patients with migraine and the clinical correlations between these abnormalities and migraine. Additionally, olfactory training is highlighted as a promising non-pharmacological treatment for migraine.

## 1. Introduction

The olfactory system detects odours critical for danger avoidance (*e.g.*, fire, spoiled food). Olfactory epithelial sensory neurons transmit odour signals to the olfactory bulb, which conveys them through the olfactory tract to the primary olfactory cortex. This cortical region subsequently projects to the secondary olfactory cortex, enabling odour perception (Fig. 1). The primary olfactory cortex comprises the anterior olfactory nucleus, piriform cortex, entorhinal cortex, and amygdala, while the secondary olfactory cortex includes the orbitofrontal cortex, insula, thalamus, and hippocampus [1, 2]. Olfactory abnormalities manifest as hyperosmia, anosmia, parosmia or hallucinations [3, 4].

**Fig. 1.** Schematic diagram of olfactory conduction. Olfactory conduction passes from the olfactory sensory neurons in the olfactory epithelium to the olfactory bulb, primary olfactory cortex, and secondary olfactory cortex. PC, piriform cortex; OFC, orbitofrontal cortex; Thal, thalamus; Hipp, hippocampus; Amyg, amygdala; Ento, entorhinal cortex; OB, olfactory bulb.

Nausea, vomiting, photophobia, and phonophobia are common symptoms of migraine [5]. However, clinical observations indicate that some patients with migraine also experience olfactory dysfunctions, such as hyperosmia, osmophobia and olfactory hallucinations. Certain irritating odours can trigger migraine attacks [6]. Although the exact pathogenesis of migraine remains unclear, the activation of the trigeminal vascular system is considered one of the primary pathogenic mechanisms [7]. Additionally, cortical spreading depression (CSD), closely associated with aura, may involve the olfactory cortex [8], leading to altered excitability in patients with migraine [9]. This narrative review aimed to explore the olfactory abnormalities in patients with migraine—a symptom often overlooked by neurologists—and analyze its clinical characteristics and potential underlying mechanisms.

## 2. Mechanisms of migraine-associated olfactory abnormalities

### 2.1 Interaction between the olfactory system and the trigeminal vascular system

The trigeminal ganglion (TG) contains pseudo-unipolar neurons whose peripheral axons innervate the meninges and dural vessels, while their central axons connect to the spinal trigeminal nucleus (SpV) in the brainstem and cervical spinal cord. Mechanical, electrical, or chemical stimulation activates meningeal perivascular nociceptors, triggering the release of vasoactive peptides that induce meningeal vasodilation and neurogenic inflammation, thereby promoting migraine development (Fig. 2). Noxious signals travel through trigeminal nerve fibers to the TG, then via central axons to the SpV, and ultimately relay to brain regions including the thalamus, parabrachial nucleus, periaqueductal gray, locus coeruleus, hypothalamus and superior salivatory nucleus (SSN). Thalamic projections to cortical areas (visual, insular, somatosensory, auditory, olfactory) may mediate migraine-related allodynia, phonophobia and osmophobia [7, 10].

**Fig. 2.** Olfactory and migraine interaction pathways. (1) Odour stimulation activates the nasal epithelial TRPA1 pathway, which in turn activates the trigeminal vascular system (orange arrow). (2) Trigeminal nervous system activation affects olfactory bulb and sensory neuron activity (white arrow). (3) Odour stimuli activate the parasympathetic nervous system through the pyriform cortex, triggering migraine attacks (green arrow). (4) Migraine attacks activate the parasympathetic nervous system and affect olfactory perception (red arrow). (5) CSD spreads to the olfactory cortex resulting in olfactory abnormalities (blue arrow). Abbreviations: M1, primary motor cortex; M2, secondary motor cortex; S1, primary somatosensory cortex; S2, secondary somatosensory; PtA, parietal association cortex; Ins, insular cortex; Au, auditory cortex; ECT, ectorhinal cortex; RS, retrosplenial cortex; V1, primary visual cortex; V2, secondary visual cortex; Thal, thalamus; Hypo, hypothalamus; PC, piriform cortex; PAG, periaqueductal gray; LC, locus coeruleus; PB, parabrachial nucleus; SSN, superior salivatory nucleus; SPG, sphenopalatine ganglion; TG, trigeminal ganglion; SpV, spinal trigeminal nucleus; OB, olfactory bulb; OE, olfactory epithelium; TRPA1, transient receptor potential ankyrin 1; Glu, glutamate; ATP, adenosine triphosphate; CGRP, calcitonin gene-related peptide; CSD, cortical spreading depression.

Odour stimulation has a bimodal effect, activating both the olfactory and trigeminal systems [11]. For example, California laurel releases the irritant umbellulone, which can trigger severe headaches [12]. Animal studies have demonstrated that the transnasal administration of transient receptor potential ankyrin 1 (TRPA1) agonists such as acrolein and umbellulone, can stimulate TRPA1 receptors in trigeminal sensory neurons located in the nasal epithelium. This stimulation induces the release of calcitonin gene-related peptide (CGRP) and activates the trigeminal vascular system, leading to headache attacks [12, 13]. Within the TG, sensory neurons innervating the nasal epithelium and meningeal vessels are located near one another. Trigeminal sensory neurons in the nasal epithelium activate the trigeminal vascular system by releasing CGRP, ATP and glutamate, which then stimulate nearby trigeminal neurons that innervate the meninges [14]. Repeated exposure of rats to acrolein for 4 days resulted in decreased periorbital mechanical nociceptive thresholds and increased c-Fos expression in the SpV caudalis [15]. Additionally, the increased meningeal blood flow observed in rats after the transnasal administration of a transient receptor potential agonist suggests that odour stimulation activates the trigeminal vascular system [16].

A functional magnetic resonance imaging (fMRI) study found that olfactory stimulation activates the rostral part of the pons, which has been implicated as a “pacing point” for migraine, further supporting a functional link between olfaction and the trigeminal system [9]. Conversely, stimulation of the trigeminal nerve also affects the olfactory perception. Branches of the trigeminal nerve innervate the olfactory epithelium and olfactory bulb and may affect odour perception. This occurs by releasing CGRP, which inhibits the olfactory sensory neuron activity in the olfactory bulb cells and olfactory epithelium [17, 18]. Moreover, the trigeminal nervous system may play a role in the development of olfactory hallucinations. The ophthalmic and maxillary branches of the trigeminal nerve, which innervate the nasal epithelium, produce burning, stinging and cooling sensations, which are consistent with the burning-like odours perceived by some patients with migraine [19].

### 2.2 Interaction between the olfactory system and the parasympathetic nervous system

Preganglionic parasympathetic neurons of the SSN activate the postganglionic parasympathetic neurons in the sphenopalatine ganglion (SPG), leading to meningeal vasodilatation, plasma protein extravasation and inflammatory molecule release (Fig. 2). This process stimulates perivascular nociceptors in the meninges, triggering migraine attacks [20]. During a migraine episode, the parasympathetic nervous system is activated by the trigeminal nucleus caudalis (TNC), resulting in nasal epithelial vasodilatation and glandular secretion. This leads to turbinate swelling, nasal congestion, and runny nose, affecting nasal patency and odour perception [21].

### 2.3 Olfactory system and cortical spreading depression

CSD is a slow-expanding depolarizing wave of neurons and astrocytes that underlies the migraine aura [10]. CSD may involve structures such as the temporal lobe, orbitofrontal cortex, and piriform cortex, resulting in olfactory abnormalities [8]. In addition to direct spread to the olfactory cortex leading to olfactory abnormalities, CSD may indirectly affect olfactory perception by activating pathways in the trigeminal system and parasympathetic nerves (Fig. 2). Additionally, CSD can induce changes in the ATP, glutamate, potassium ions and hydrogen ion concentrations, along with the localized release of CGRP and nitric oxide from activated perivascular nerves (Fig. 2). These changes trigger the meningeal vascular injury receptors, which in turn activate the trigeminal vascular system and affect olfactory perception [10].

### 2.4 Altered olfactory cortex excitability in migraine

An ^15^O-labeled water (H_2_^15^O)-positron emission tomography (PET) study revealed increased cerebral blood flow in the left piriform cortex and anterosuperior temporal gyrus in patients with migraine and hyperosmia, indicating heightened olfactory cortex excitability in these individuals [22]. 4-(2′-methoxyphenyl)-1-[2′-(N-2′′-pyridinyl)-p-[^18^F]fluorobenzamido]ethylpiperazine ([^18^F]MPPF) is a selective 5-hydroxytryptamine 1A (5-HT_1A_) receptor antagonist. An [^18^F]MPPF-PET study found that odour stimulation induced a significant increase in the [^18^F]MPPF binding potential in the left orbitofrontal cortex, precentral gyrus, and temporal pole of patients with migraine and hyperosmia [23]. Similarly, an fMRI study confirmed that the amygdala, insular cortex, temporal pole, superior temporal gyrus, and cerebellum exhibit higher blood oxygen level-dependent signals during olfactory-stimulated migraine attacks [9]. This increased cortical excitability during acute attacks may partially explain the occurrence of symptoms such as osmophobia, olfactory hallucinations and hyperosmia in patients with migraine.

## 3. Characteristics of migraine-associated olfactory abnormalities

### 3.1 Migraine attacks triggered by odour stimulation

Odour triggers were present in 30.1%–78.2% of patients with migraine. A migraine attack may be triggered by exposure to a particular odour, which can occur multiple times, with just a few minutes of exposure potentially provoking an attack [11, 24, 25]. The common odour triggers include fetid odours, cooking products, oil derivatives, shampoos and conditioners, cleaning products, perfumes, insecticides, and roses. Among cleaning products, floral-scented hair-styling preparations, laundry detergents and fabric softeners are notable [11]. Perfumes, cigarette smoke, and cleaning products are the most common triggers [6].

Although odour stimulation has low sensitivity, it has high specificity as a migraine trigger and helps differentiate migraine from other primary headaches [24]. Migraine triggers, such as odours, are significantly associated with pain intensity, duration and accompanying symptoms, including vomiting and phonophobia, particularly in children and adolescents [26]. Odour triggers and osmophobia are more common in women, which may be related to more pronounced odour-induced cortical activation in women [27]. Female patients with migraine with odour triggers tend to experience an earlier onset and longer disease duration [28]. However, a recent study found no differences in age, sex, or migraine type between patients with and without odour-triggered migraines [11]. This discrepancy may stem from variations in sex, age distribution, migraine type and comorbidities among the studied patients.

### 3.2 Osmophobia

Osmophobia refers to the aversion, fear, and intolerance to odours, even those that are pleasant [29]. It has also been reported in patients with migraine and tension-type headache (TTH) [30]. Osmophobia is more common in female migraine patients [31]. It can occur not only during a migraine attack but also during the interictal phase. In more than half of patients, migraine attacks are accompanied by osmophobia; in more than two-thirds of patients, osmophobia occurs simultaneously with a migraine attack [32].

The prevalence rates of osmophobia were 25.1%–86% during migraine attacks and 24%–53.3% during the interictal phase [33]. Patients with migraine, and osmophobia are more likely to experience a variety of symptoms, including anxiety, depression, vomiting, and more severe and prolonged headaches [26, 34, 35]. Osmophobia often follows a certain pattern, with the same odour type recurring during different headache episodes [34]. It may serve as an early marker of migraine. In a prospective study of juvenile patients with TTH, 22.6% without osmophobia and 62.2% with osmophobia were later diagnosed with migraine after 3 years [36].

Osmophobia has low sensitivity but high specificity for diagnosing migraine, making it a valuable tool for differential diagnosis and potentially a diagnostic criterion for migraine in the future [26, 34, 37]. Patients with migraine and osmophobia tend to experience more severe headache, insomnia, fatigue, anxiety, and depression and are at a higher risk of suicide [38, 39, 40].

### 3.3 Olfactory hallucinations

Olfactory hallucination refers to the perception of odours in the absence of any actual odour source [4]. These hallucinations can occur in patients with epilepsy, Parkinson’s disease, neoplastic diseases, or infectious diseases. In patients with migraine, olfactory hallucinations often involve a variety of odours, predominantly unpleasant ones, such as cigarettes, garbage, food and burning odours. These odours are typically distinctive and recognizable [8, 41].

The prevalence rates of olfactory hallucination are approximately 0.1% in adults and 3.9% in children [41, 42]. Most olfactory hallucinations occur before or during headache attacks, although some may occur without a concurrent headache [43, 44]. These hallucinations share aura-like characteristics, are completely reversible and usually last from minutes to hours [8, 41]. Given these features, further investigation is needed to determine whether olfactory hallucinations should be included as an aura symptom in the diagnostic criteria for migraine with aura.

### 3.4 Hyperosmia

Hyperosmia refers to an increased sensitivity to odours. A questionnaire-based survey of 113 patients with migraine found that 38.1% experienced heightened sensitivity to odours before their headache attacks, whereas 61.9% experienced it during the attacks [25]. Patients with migraine and hyperosmia during the interictal phase have a longer disease duration, which correlates with the migraine disability assessment score [25]. Patients with migraine and hyperosmia during the interictal phase were assessed using an oral questionnaire and a chemical odour intolerance index [45].

Hyperosmia was reported in 35.2% of patients with migraine. Migraine attacks were more frequent in patients with hyperosmia, who were also more likely to experience odour-triggered migraine attacks [45]. Interictal hyperosmia, assessed using a questionnaire and visual analog scale, was identified in 14% of patients with migraine. Patients without interictal hyperosmia are more likely to experience osmophobia and odour-induced migraine [6]. Hyperosmia does not necessarily reflect a decreased olfactory threshold but is also related to the ability of patients with migraine to identify odours. A decreased ability to identify specific odours may cause patients to perceive themselves as being sensitive to all odours [46].

### 3.5 Hyposmia/anosmia

Hyposmia or anosmia refers to the decreased ability or inability to perceive odours [4]. Patients with migraine were found to have lower olfactory identification rates, assessed using the Brief Smell Identification Test [47]. When evaluated using the Sniffin’ Sticks test, patients with migraine were found to have impaired odour recognition, with those experiencing allodynia demonstrating a poorer ability to identify odours [48]. Patients with migraine have lower olfactory threshold, discrimination, and identification scores on the Sniffin’ Sticks test, with these impairments being more pronounced in those with osmophobia [46]. Similarly, some patients with migraine exhibit hyposmia or anosmia during acute attacks, as assessed using the University of Pennsylvania Smell Identification Test [49]. A study examining trigeminal and olfactory event-related potentials (ERPs) in 19 patients with migraine and 19 controls, using CO_2_ and hydrogen sulfide (H_2_S) stimulation, found that patients with migraine had stronger responses to trigeminal stimulation, with significantly greater N1 wave amplitudes. The olfactory ERP amplitudes were smaller, indicating that patients with migraine experienced hyposmia [50]. In addition to electrophysiological findings, an MRI study found that patients with migraine, particularly those with osmophobia, had reduced bilateral olfactory bulb volumes. A decreased olfactory bulb volume is associated with anosmia, providing imaging evidence of olfactory dysfunction in these patients [51].

A threshold test using pyridine conducted in 67 patients with migraine identified hyposmia or anosmia in 18% of the patients [52]. A separate test using vanillin and acetone found that patients with migraine had reduced olfactory thresholds for vanillin, but no difference was observed for acetone [53]. Both adult and pediatric patients have shown high olfactory threshold scores and hyperosmia [48, 54]. Odour identification testing is commonly used in research and clinical practice owing to its simplicity and time efficiency. However, the olfactory threshold test is preferred when examining olfactory function in patients with headache, as it focuses solely on peripheral olfactory system sensitivity. Meanwhile, odour identification and discrimination involve the ability to comprehend, learn, and remember [54].

## 4. Olfactory training: a nonpharmacologic treatment for migraine

Olfactory training involves systematic exposure to odour stimulation over a specified period [55]. The currently accepted olfactory training protocols include: choosing 3–4 scents (*e.g.*, rose, peach, orange, lavender, apple, lemon, cinnamon, strawberry, caramel, chocolate, and clove) based on personal preference. Participants underwent twice-daily (morning and evening) olfactory exposure to pleasant odours, with each session comprising approximately 10–20 seconds of controlled odour presentation per stimulus. The intervention protocols were maintained over a three-month period [56, 57]. This practice improves olfactory function, regulates mood, and enhances cognition [58]. Additionally, olfactory training increases chronic pain thresholds in adult patients, suggesting a potential role in pain relief through desensitization [59]. A study involving 80 children and adolescents with migraine or tension-type headache found that olfactory training significantly increased headache thresholds, decreased headache frequency, reduced headache-related disabilities and improved mood and sleep quality [57]. Neuroanatomical convergence exists between olfactory processing pathways and migraine-associated networks at the central level, particularly within limbic-cortical circuits involving the insular cortex, anterior cingulate, hippocampal formation and amygdaloid complex. Olfactory training may induce neuroplastic adaptations manifesting as both structural reconfiguration and functional connectivity modulation within nociceptive processing hubs [56]. As such, olfactory training emerges as a valuable non-pharmacological treatment for migraine.

## 5. Limitation analysis

The mechanistic interplay between migraine and olfactory abnormalities remains scarcely investigated, with existing literature predominantly reliant on clinical observations. There may be differences in olfactory abnormalities among various migraine subtypes. Furthermore, the assessment of olfactory abnormalities remains primarily reliant on subjective patient-reported experiences, with a notable absence of robust quantitative methodologies in this research domain.

## 6. Conclusions

Patients with migraine may present with various forms of olfactory dysfunction, such as hyperosmia, osmophobia, olfactory hallucinations, hyposmia, or even anosmia. The olfactory system interacts with the trigeminal and parasympathetic nervous systems. CSD affects the olfactory cortex, leading to altered excitability. Patients with migraine and osmophobia were more likely to experience anxiety, depression, and vomiting. Osmophobia demonstrates low sensitivity but high specificity for diagnosing migraine, making it beneficial for differential diagnosis. Future studies should consider including osmophobia as a diagnostic criterion for migraine. Most olfactory hallucinations occur prior to a migraine attack, but some patients may experience them without a headache. Patients with migraine and hyperosmia tend to have more frequent migraine attacks and a higher likelihood of odour-induced migraine attacks. They may present with hyposmia or anosmia, as evidenced by a reduction in the bilateral volume of the olfactory bulbs. Finally, olfactory training is a valuable non-pharmacological treatment for migraine.
